# Microfluidic Chips for *In Vivo* Imaging of Cellular Responses to Neural Injury in *Drosophila* Larvae

**DOI:** 10.1371/journal.pone.0029869

**Published:** 2012-01-23

**Authors:** Mostafa Ghannad-Rezaie, Xing Wang, Bibhudatta Mishra, Catherine Collins, Nikos Chronis

**Affiliations:** 1 Department of Biomedical Engineering, University of Michigan, Ann Arbor, Michigan, United States of America; 2 Department of Molecular, Cellular, and Developmental Biology, University of Michigan, Ann Arbor, Michigan, United States of America; 3 Department of Mechanical Engineering, University of Michigan, Ann Arbor, Michigan, United States of America; VIB & Katholieke Universiteit Leuven, Belgium

## Abstract

With powerful genetics and a translucent cuticle, the *Drosophila* larva is an ideal model system for live imaging studies of neuronal cell biology and function. Here, we present an easy-to-use approach for high resolution live imaging in *Drosophila* using microfluidic chips. Two different designs allow for non-invasive and chemical-free immobilization of 3^rd^ instar larvae over short (up to 1 hour) and long (up to 10 hours) time periods. We utilized these ‘larva chips’ to characterize several sub-cellular responses to axotomy which occur over a range of time scales in intact, unanaesthetized animals. These include waves of calcium which are induced within seconds of axotomy, and the intracellular transport of vesicles whose rate and flux within axons changes dramatically within 3 hours of axotomy. Axonal transport halts throughout the entire distal stump, but increases in the proximal stump. These responses precede the degeneration of the distal stump and regenerative sprouting of the proximal stump, which is initiated after a 7 hour period of dormancy and is associated with a dramatic increase in F-actin dynamics. In addition to allowing for the study of axonal regeneration *in vivo*, the larva chips can be utilized for a wide variety of *in vivo* imaging applications in *Drosophila*.

## Introduction

Axons are vulnerable components of neuronal circuitry, hence it is of great interest to understand how neurons respond to axonal injury. The ability of an axon to regenerate requires an environment favorable to axonal growth, as well as a series of intrinsic cellular events that occur on different time scales after injury [1]–[3]. Within seconds of injury, intracellular levels of calcium rapidly rise and then decay, and this wave of intracellular calcium appears to be an important precursor to subsequent regeneration [4]–[6]. Later responses include a transcriptional response facilitated by an increase in cAMP [7], the retrograde transport of signaling molecules [8], and a delivery via anterograde transport of new cellular components into the axon [9], [10]. Concomitantly, the distal stump, which has been disconnected from the cell body, undergoes Wallerian degeneration [11] clearing the way for regenerating axon. Importantly, axonal regeneration requires the formation of a functional growth cone from the injured proximal stump. A characteristic feature of the growth cone is its highly dynamic and coordinated structure of filamentous actin, which allows it to change shape in response to cues in the environment.

Recently the development of various laser ablation techniques [12] in combination with microfluidic technology [13] has enabled the *in vivo* monitoring of regenerative responses to axonal injury in the nematode *C. elegans* [14]–[18]. However, *C. elegans* neurons display some behaviors not observed in vertebrate neurons, including re-fusion of the broken axonal fragments [13], [19], [20].

Like *C. elegans*, *Drosophila* larvae have a translucent cuticle and a simple neuroanatomy that is amenable to *in vivo* imaging [5], [21]. Moreover, axons in the *Drosophila* peripheral nervous system are ensheathed by glial cell membranes [22], [23] and undergo Wallerian degeneration similarly to vertebrate axons [24]–[26]. *Drosophila* axons are also capable of undergoing new axonal growth after injury, and genetic studies indicate that conserved signaling molecules are required for this process [24], [27].

Despite the great potential, several technical issues need to be addressed in order to perform *in vivo* imaging in *Drosophila*. For high resolution imaging of rapid events such as calcium waves and axonal transport, the animal needs to be exceptionally stationary during immobilization. On the other hand, time lapse imaging of long term events such as new axonal growth requires a gentle immobilization technique which will not interfere with the physiology of the animal. Conventional immobilization approaches involve dissection [28], [29] or the use of chloroform [30]. Isofluorane is also used as an anesthetic for long-term, time-lapse imaging [21], [31]. While the use of anesthetics has many advantages, anesthetics are known to inhibit neural activity and alter neural physiology [32]–[34]. Because the larva can survive only short doses of the chemical, time-lapse imaging must be restricted to time intervals of 2 hours in order to allow recovery between doses of the anesthetic [21], [32], [33]. In addition, human safety concerns must also be taken into account when working with conventional anesthetics. In order to image cellular events over broader timescales and with higher throughput, a chemical-free immobilization method is needed.

Here, we report a microfluidic-based immobilization methodology for time-lapse imaging in *Drosophila* larvae. Our approach employs mechanical forces and/or supply of carbon dioxide gas (CO_2_) in order to temporally immobilize single 3^rd^ instar larva. It minimizes the stress on the larva body (recovery takes place in less than 30 seconds) and allows repetitive *in vivo* imaging to be performed over extended periods of time. The proposed microfluidic ‘larva chips’ can be efficiently utilized for imaging various cellular events which occur on different time scales, ranging from milliseconds up to several hours. While in this work we focus upon cellular responses to targeted injury of neurons, the microfluidic chips can be broadly used for *in vivo* imaging of many different processes in *Drosophila* larvae.

## Results

### The SI and LI microfluidic larva chips

**Figure 1:**
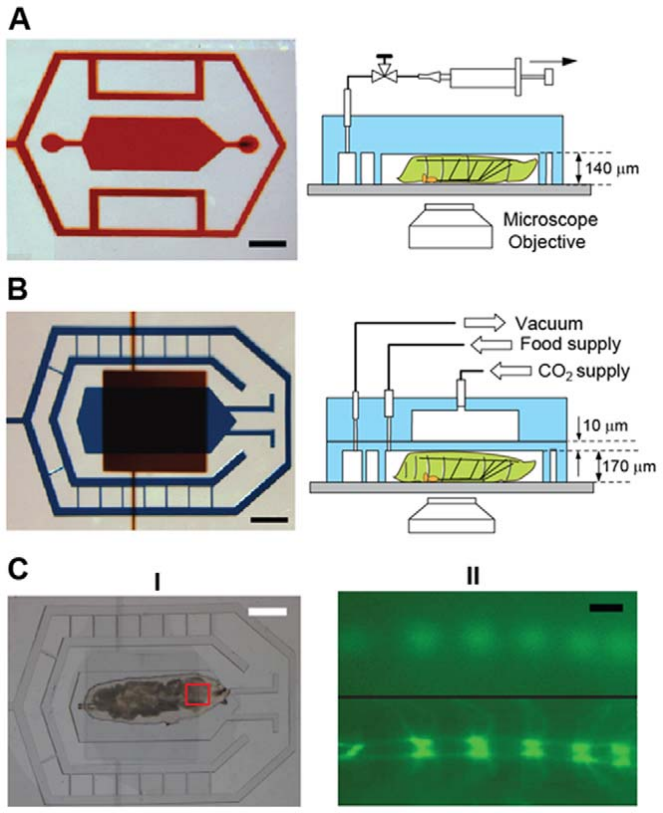
The SI and LI microfluidic chips for immobilizing *Drosophila* larva. (A) The SI-chip is a single-layer PDMS microfluidic device that utilizes a shallow (140 µm thick) immobilization microchamber to fix a 3^rd^ instar larva in the vertical direction. Scale bar, 1 mm. (B) the two-layer LI-chip. The first PDMS layer (labeled with blue color) has the larva immobilization microchamber and is connected to two microfluidic channels to supply food to the larva head (typically delivered every 30 min). A second PDMS layer (labeled with red color) is vertically integrated into the first PDMS layer to deliver CO_2_ through a 10-µm thick PDMS membrane. In both the SI and LI chips, a microfluidic network surrounding the immobilization chamber is used to create a tight seal between the PDMS and the glass coverslip. Scale bar, 1 mm. (C) (I) Bright-field image of a 3^rd^ instar larva immobilized in the LI-chip. Scale bar, 1 mm. (II) Fluorescent images of the larva body (highlighted in the red square in C(I)) before (top image) and after (bottom image) immobilization. After application of CO_2_ at 5 psi, the larva is immobilized and the GFP-labeled ventral nerve cord is brought into focus (bottom image). Scale bar, 20 µm.

In order to perform axonal injury and to study the resulting cellular responses *in vivo*, we designed two PDMS (polydimethylsiloxane) microfluidic devices (Figure 1): one for short-term (up to 1 hour) and one for long-term (up to 12 hours) immobilization of *Drosophila* larvae. Both devices are reversibly attached to a glass coverslip. The first microfluidic device, termed the ‘SI-chip’ (for *S*hort-term Immobilization), contains a 3.5 mm long, 1.5 mm wide and 140 µm thick micro chamber (the ‘immobilization micro chamber’) which is designed to snugly fit the body of an early-stage 3^rd^ instar larva (which is approximately 200 µm thick), The micro chamber is surrounded by a microfluidic network that is held under constant vacuum in order to maintain a strong seal between the PDMS, oil, and coverslip interface. The mild pressure applied to the larva body through the PDMS/glass walls is sufficient to completely immobilize it. The pressure brings internal body structures, such as segmental nerves which contain motoneuron and sensory neuron axons, close to the coverslip, allowing for high-resolution imaging through the use of high numerical aperture microscope objectives. After release of the vacuum, the larva can be easily removed from the micro chamber, allowing additional experiments to be performed. This purely mechanical immobilization approach can keep >90% of larvae alive for continuous immobilization periods of up to 1 hour (Figure S1A).

To increase the survival rate over longer (>1 hour) periods of time and therefore enable long-term imaging, we designed a second PDMS microfluidic device (Figure 1B, 1C), termed the LI-chip (for Long-term Immobilization). The LI-chip has double-layer architecture, incorporating a ‘CO_2_ micro chamber’ for delivering a mixture of CO_2_/air to completely immobilize the larva body. The CO_2_/air mixture is supplied to the chip through a 95/5% (CO_2_/air) gas tank. The CO_2_ micro chamber is integrated on top of a 170 µm thick immobilization micro chamber, which is dimensionally similar to the SI-chip, through a 2-layer micro fabrication approach [35]. The two microchambers are separated by a 10 µm thick PDMS membrane through which CO_2_ can diffuse into the immobilization micro chamber. CO_2_ is supplied under moderate pressure (5 psi) to the LI-chip resulting in the deflection of the PDMS membrane that collapses onto the larva body. This dual (mechanical and CO_2_-based) immobilization approach has an additional advantage over the SI-chip: when the CO_2_ supply is turned off and the chamber is flushed with air, the larva can be held comfortably in an semi-immobilized state, stretching and contracting against the PDMS membrane without leaving the field of view. Larvae become motile within 30 seconds after the CO_2_ supply is turned off, even after many successive immobilization bouts (every 5 minutes over 10 hours) (Figure S2). To avoid starvation, the LI-chip is also equipped with two microfluidic channels to allow for food supply. Using this dual immobilization approach, larva can be kept alive on-chip for more than 10 hours (Figure S1B, S1C).

### On-chip calcium imaging within milliseconds of injury

**Figure 2:**
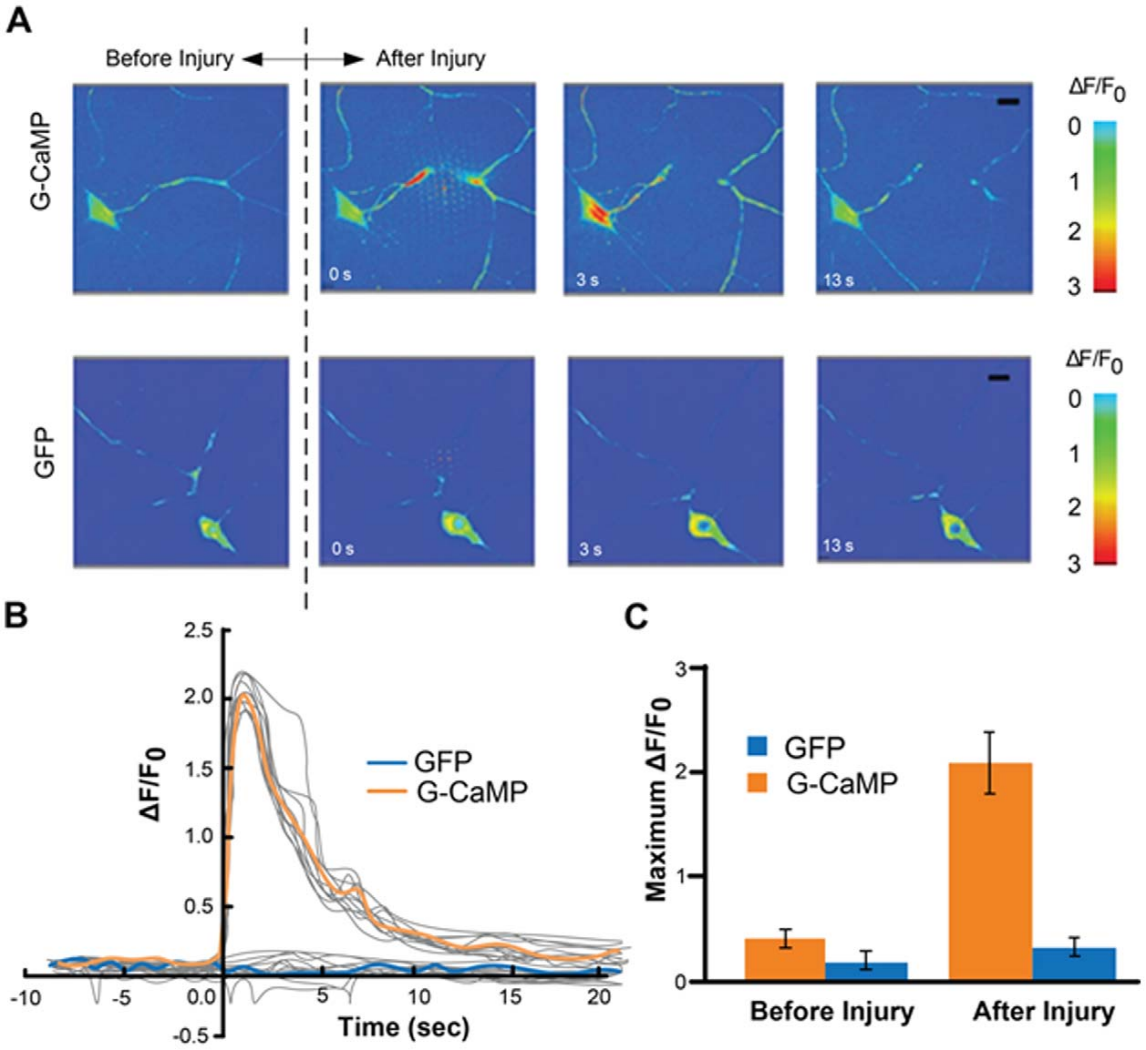
Calcium dynamics after laser microsurgery. (A) Time-lapse images from immobilized larvae depict intracellular calcium dynamics during laser microsurgery of a sensory neuron dendrite, using a pulsed UV laser (see also Movies S1 and S2). The ppk-Gal4 driver was used to express UAS-G-CaMP3.0 or UAS-mCD8-GFP. (B) Average normalized fluorescent intensity (ΔF/F_0_) was calculated in the cell bodies for G-CaMP3.0 and mCD8-GFP (sample size, n = 12) before and after injury (injury is performed at 0 sec). A peak value in the intensity of G-CaMP is observed 2 seconds after injury. Calcium transients from individual larvae are depicted in light grey color. (C) Quantification of the maximum fluorescent intensity change (maximum ΔF/F_0_). The fluorescent intensity from mCD8-GFP expressing neurons did not change significantly (p-value<0.01, n = 12).

The SI-chip enabled us to perform laser axotomy and measure rapid changes in intracellular calcium after injury. To detect intracellular calcium, we used the genetically encoded calcium sensor G-CaMP 3.0 [36] which was expressed in Class IV sensory neurons via the Gal4/UAS system (Figure S3). After immobilizing single larva in the SI-chip, a pulsed UV dye laser was used to transect a single dendrite [37]. Injury induced an instant and dramatic increase at the G-CaMP fluorescence level at the injury site, which rapidly spread along both the proximal and distal compartments of the dendrite (Figure 2A, Movie S1). Within 2 seconds after injury, a ∼200% increase in fluorescence (ΔF/F_0_≈2, where F_0_ is the baseline fluorescence level before injury) was detected in the cell body, which returned to baseline levels within 15 seconds (Figure 2B). For comparison, mCD8-GFP expressed in the same neurons, did not yield a significant change in intensity after laser axotomy (Figure 2B, 2C, see also Movie S2). The kinetics of the observed Ca^2+^ responses resemble Ca^2+^ transients after axonal injury in other model organisms [5], [6], [38], [39]. The SI-chip can therefore be used to perform *in vivo* laser microsurgery on-chip as well as to quantify the resulting responses.

### Monitoring changes in axonal transport after injury

Microtubule-based motors, kinesins and dynein, are known to carry vesicles and organelles at rates of 0.1–5 µm/sec in axons [40], [41]. Documentation of this motility requires rapid imaging with high magnification, high numerical aperture objectives, and precise immobilization of the larva. Dense core synaptic vesicles, labeled by expressing ANF-GFP [42] specifically in aCC and RP2 motoneurons using the Gal4/UAS system, were imaged in intact larvae using the SI-chip (Movie S3) and in dissected ‘flay-open’ larvae as previously described [24]. Image analysis revealed that the overall density of anterogradely moving, retrogradely moving, and stationary particles (particles/100 µm of axon length) did not change significantly (Figure S4). We conclude that the SI-chip is an effective method for imaging and measuring properties of axonal transport, equivalent to the ‘flayed open’ approach.

**Figure 3:**
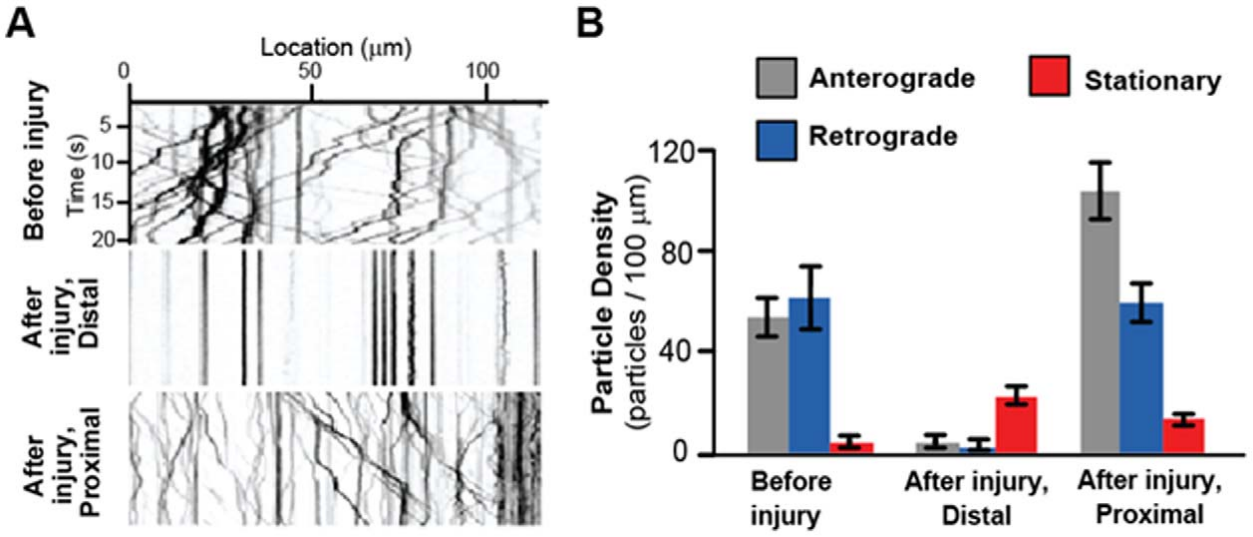
Changes in axonal transport after nerve-crush injury. (A) Kymographs from example movies of ANF-GFP labeled vesicles in an uninjured axon (Movie S3), the distal stump 3 hours after injury (Movie S4) and the proximal stump (Movie S5) 3 hours after injury. (B) Particle density (anterograde, retrograde, and stationary) was quantified per 100 µm of axon length. In the proximal stump 3 hours after injury, there was a 90% increase in anterograde particle density (p-value = 0.03, n = 16), but no significant change in retrograde particle density (p-value<0.01, n = 16). In contrast, transport was almost completely halted in the distal stump (p value<0.01, n = 8). Error bars represent standard error of the mean.

We investigated whether injury, introduced by nerve crush [24], alters the properties of axonal transport. To do this, we analyzed the motility of ANF-GFP particles in aCC and RP2 motoneuron axons for several time points after injury (0, 1, 3, 5, 7 and 9 hours). Crush-injured larvae were immobilized in the LI-chip for ∼30 sec in order to obtain images for each time point. We observed dramatic changes in axonal transport at both the distal and proximal axonal stumps within a surprisingly short period (3 hours) after injury (Figure 3A). Within the distal stump, nearly all of the ANF-GFP particles were immotile within 3 hours after injury (Movie S4). In contrast to the immobility in the distal stump, we observed a 90% increase in the proximal stump anterograde particle density 3 hours after injury (Figure 3B, Movie S5). While the cessation of transport in the distal stump may be a precursor to Wallerian degeneration, the increase in transport in the proximal stump may be important for new axonal growth.

### Long term time lapse imaging of axonal sprouting after injury

**Figure 4:**
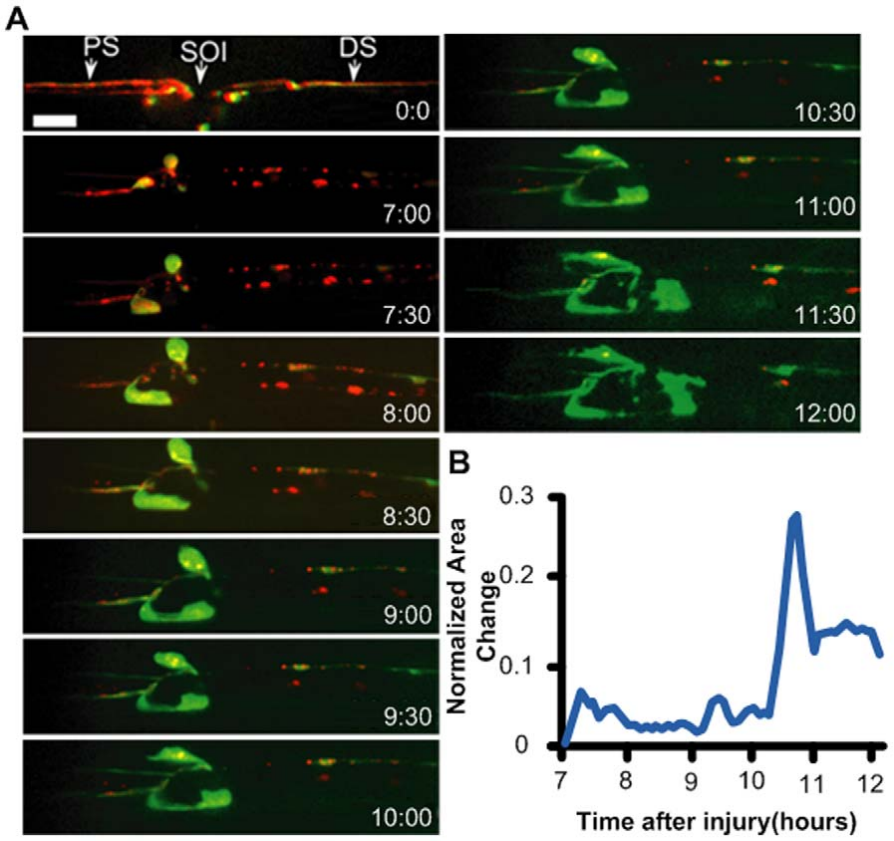
Axonal sprouting after laser injury. (A) *In vivo* time-lapse images of the proximal stump from 7 hours to 12 hours after laser axotomy. A single enteric motoneuron was visualized by combining the RN2-Gal4_ENREF_48 [48] driver line with UAS-mCD8-RFP (red) to label axonal membrane, and UAS-GFP-moesin [54] (green) to label F-actin. The proximal site (PS) of injury, the site of injury (SOI), and the distal site (DS) of injury are highlighted right after injury (0∶00 frame). Scale bar, 10 µm. (b) Normalized area change of the proximal stump over time. Significant movement in the proximal stump is observed ∼10.5 hours after injury.

The ideal method for studying and quantifying regenerative axonal growth is to conduct longitudinal time-lapse imaging of single axons after injury. We tracked the proximal and distal stumps of an injured axon by collecting high-resolution confocal images throughout a 5-hour (7–12 hours) time course (Figure 4, Movie S6). We observed that the proximal stump is relatively dormant for the first seven hours after injury. However between 7 and 12 hours after injury new axonal sprouting can be readily observed. To visualize F-actin we expressed a GFP-tagged version of the F-actin binding protein moesin [43]. By quantifying the area change of the proximal stump of the injured axon (Figure 4B), we noted that F-actin is particularly dynamic between 10 and 11 hours after injury (Figure S5). These observations highlight the use of the larval chips to study dynamic events *in vivo* over long periods of time.

## Discussion

### The SI and LI chips for *Drosophila* larva immobilization

The use of microfluidic chips for larva immobilization has several advantages over conventional approaches: (i) microfluidics replace the use of chemicals which alter neural physiology, allowing for *in vivo* imaging of unanesthesized animals, (ii) larvae do not need a recovery period after immobilization, allowing for imaging over a broad range of time scales, (iii) the immobilization conditions are reproducible and well-controllable (iv) the microfluidic chips are simple to fabricate and their design can be easily adapted to immobilize larvae of different developmental stages or many larvae at once. While we described in this report the use of microfluidics for studying responses to neural injury, we envision that the larva chips can be broadly used to study many different cellular events *in vivo*. Examples include the formation of new synaptic contacts at neuromuscular junctions, the motility of cytosolic components in neurons, muscles, or glia, and calcium signaling within individual cells as an indicator of neural activity.

The chemical free larva chips provide an attractive alternative to previous methods of immobilizing larvae for imaging [21], [30], [31]. While use of isofluorane can immobilize the animal more completely, halting the heartbeat, this method cannot be used to monitor physiology, and requires two hours of recovery time between imaging sessions [21], [31]. Our behavioral data suggest that the use of CO_2_ in the LI chip has a minimum impact on the larva: (i) the survival rate and average body movement are not affected after 10 hours of repetitive CO_2_ application (Figures S1B and S1C), and (ii) the recovery time after 10 hours of repetitive CO_2_ immobilization is minimally affected (Figure S2B). Moreover, the SI-chip allows for imaging in the absence of CO_2_.

### The SI chip allows for *in vivo* imaging of neural activity

The non-invasive, purely mechanical approach for immobilizing live larvae in the SI chip creates an ideal environment for measuring intracellular Ca^2+^ dynamics *in vivo*. We observed that laser microsurgery of a single neurite induced a wave of Ca^2+^ similar to injury in other model organisms [5], [6], [38], [39]. This method, coupled with well-established genetic approaches in *Drosophila*, will allow for a better understanding of the role of Ca^2+^ dynamics in axonal regeneration and degeneration processes after injury. Furthermore, this demonstrates that the SI chip can be used for rapid imaging of intracellular Ca2+ dynamics in the intact animal.

### The larva chips allow monitoring of rapid changes in axonal transport

The SI and LI microfluidic chips were particularly useful for studying changes in axonal transport after injury, which we were unable to study over long periods of time in dissected animals. We observed rapid and asymmetric changes in axonal transport in proximal and distal sides of the injury, with an increase in anterograde transport in the proximal stump, and a cessation of all transport in the distal stump. This dichotomy on either side of the injury site correlates with the opposite outcomes: the proximal stump of the aCC and RP2 neurons initiate new axonal growth and branching, while the distal stumps undergo Wallerian degeneration [24].

The cessation of transport throughout the entire distal stump within 3 hours of injury parallels previous observations in mice that mitochondrial transport in the distal process halts within a similar time frame [44]. Interestingly, fragmentation of the axonal membrane and microtubules for these neurons does not begin until about 6 hours after injury (X. Xiong and C. Collins, personal communication), so the cessation of transport and may represent an early stage in the process of Wallerian degeneration. Because transport halts throughout the entire distal process, it may involve a rapidly diffusing cue, such as calcium, from the injury site. In contrast to the distal stump, the number of anterogradely moving particles increases in the proximal stump within 3 hours of injury. This increase in axonal transport parallels observations in vertebrate peripheral axons and may play an important role in promoting new axonal growth [9], [10].

### Using larva chips to study axonal regeneration *in vivo*

Time lapse imaging is an essential tool for studying changes in cell morphology over long periods of time. Microfluidics techniques have previously enabled the study of axonal regeneration after injury *in C. elegans* [13]. However *C. elegans* neurons display some behaviours that are seldom observed in vertebrate neurons, For instance, *C. elegans* axons can regrow along the distal stump, and can sometimes re-fuse with the distal stump [15], [16], [19]. In contrast, our observations indicate that *Drosophila* axons behave more similarly to vertebrate axons. While the proximal stump undergoes extensive sprouting it never makes contact with the distal stump, and may indeed be repelled by the distal stump [45], [46].

Using the larval chips, we observed that the proximal stump begins to undergo profound morphological changes after a period of dormancy. The minute-to-minute changes in F-actin structure suggest the existence of a dynamic network of actin, which would be a fundamental component of a functional growth cone. It is intriguing that the dynamics are not observed immediately after injury, but require at least 7 hours after injury to be initiated. Previously characterized transcriptional responses to injury require a similar time course in *Drosophila* neurons [24], so this time of dormancy may reflect the need for new gene expression or transport of new material in order to form a new growth cone. In future studies, it will be interesting to determine the cellular requirements for initiation of those dynamics, using *Drosophila* larva as a genetically tractable model system.

## Methods

### Chip microfabrication

Master molds were microfabricated by spinning and patterning SU-8-2050 photoresist on silicon wafers. To generate the SI-chip, a 10∶1 polydimethylisiloxane (PDMS) prepolymer mixture was cured in a 140 µm-thick SU-8 mold for 4 hours at 65°C. For the LI-chip, a similar procedure was used to generate two SU-8 molds. The first mold was 170-µm thick, the second one was 100-µm thick. To fabricate the first PDMS layer of the LI-chip, a 1∶15 PDMS mixture was spin cast at 415 rpm and cured over the first SU-8 mold, resulting in a 180-µm thick PDMS layer. A second PDMS slab that contained the CO_2_ microchamber was microfabricated by casting and curing a 10∶1 PDMS mixture over the 100-µm thick SU-8 mold and plasma bonded into the first PDMS layer. Fluidics inlets/outlets outlets were punched into the PDMS slabs using a sharpened, 19-gauge stainless steel needle.

### Larva loading

Single early stage 3^rd^ instar larvae (∼4 mm in length) were immersed in halocarbon oil 700 (cat. # H8898 Sigma Aldrich Inc.), placed on a glass coverslip, and then manually aligned and covered with the SI- or LI-chip. A tight seal between the PDMS chip and the coverslip was created by applying weak vacuum (600 mTorr) to the outlet of the microfluidic network. Vacuum was generated: (i) manually, using a 20 cc syringe in the SI-chip, and (ii) using a mechanical pump in the LI-chip. CO_2_ was supplied at the top PDMS layer of the LI-chip at 5 psi.

### Imaging

All *in vivo* imaging recordings were conducted using a spinning disk confocal system (Perkin Elmer), consisting of a Yokagawa Nipkow CSU10 scanner, and a Hamamatsu C9100-50 EMCCD camera, mounted on a Zeiss Axio Observer with 63× (1.5 NA) oil objective. Volocity software (Perkin Elmer) was used for all image acquistion and analysis.

### Injury assays

Laser injuries were performed using a nanosecond 435 nm pulsed UV dye laser (Photonic instruments Inc.), as described previously [37]. Nerve crush injuries were performed by pinching the dorsal part of the animal containing segmental nerves with Dumostar number 5 forceps [24].

### Calcium imaging

We expressed UAS-GCaMP3.0 [36] with ppk-Gal4 in class IV sensory neurons [47]. Videos were captured at ∼3 frames per second (300 msec exposure time). The cell body average fluorescence signal was then extracted from each frame and background-corrected using Volocity software (Perkin Elmer).

### Axonal Transport

RRa(eve)-Gal4 was used to drive expression of UAS-ANF-GFP specifically in aCC and RP2 motoneurons [28], [48]. Segmental nerves were imaged continuously at ∼5 frames/sec (exposure time was set to 200 milliseconds). To generate kymographs, the collection of single frames spanning one minute of imaging time were processed using the ‘Multiple Kymograph’ plug-in for ImageJ [49]. For analysis, we used a MATLAB program to automate particle detection and tracking from kymographs. The program uses fast reconstruction techniques and line tracking methods, along with manual correction, to delineate individual particle traces. First, the program searched for particles in the kymograph image using regularized Hough transform [50] and then tracked each particle using B-Snake method [51]. The program then removed the trace of the detected particle from the kymograph image and the paricle tracking step was repeated until no further particles were found. The trace of each identified particle was then verified and corrected manually. From each trace, many independent properties, including segment velocities and particle density, could be measured.

### Analysis of axonal regeneration

Changes in the proximal stump structure were quantified from time-lapse confocal images collected at a sampling rate of one z-stack of images per min. First, movement artifacts between frames were reduced using a fast block matching algorithm [52]. For each frame, the area of the proximal stump was generated using a B-Snake method [51]. The non-overlapping area between two subsequent frames, representing the area change of the proximal stump, was then calculated. Finally, the area change was normalized with respect to the area of the proximal stump in each frame.

### Fly genetics

The following strains were used: RRa-Gal4 [48], RN2-Gal4 [48], UAS-G-CaMP3.0 [36], UAS-mCD8-GFP [53], UAS-mCD8-RFP, UAS-ANF-GFP [42], UAS-GMA [54].

## Supporting Information

Figure S1 Survival rates and body movement of on-chip immobilized larvae. (A) Survival rate of continuously immobilized larvae using the SI-chip. Five different immobilization microchamber thicknesses were tested. We considered a thickness of 140 µm (red curve) to be optimal, as thicknesses higher than 140 µm resulted in poor immobilization. (B) Survival rates on the LI-chip using periodic immobilization (30 s of immobilization every 5 min). We considered a thickness of 170 µm (red curve) to be optimal as more than 85% of larvae survived the immobilization procedure after 10 hours. (C) Average larva body movement using the LI-chip for different thicknesses of the immobilization microchamber (30 s of immobilization every 5 min). When the mechanical Immobilization was minimized (e.g. using a 200 µm thick microchamber), the average body movement was not affected even after 10 hours of repetitive CO_2_ application. In all plots, error bars represent standard error of the mean obtained from 10 larvae.
(TIF)

Figure S2 Larva recovery after immobilization on-chip. (A) Larva average body movement before and after the initial immobilization. The red line is the average movement from 10 larvae. The grey lines represent movement from individual larvae. The dashed line represents the immobilization period (30 sec). We define the recovery time as the time needed for the average body movement to reach the pre-immobilization value. (B) Recovery time at the beginning (0 hours) and end (10 hours) of a 10-hour repetitive CO_2_ immobilization experiment (30 sec CO_2_ immobilization/5 min resting interval). Error bars represent standard error of the mean. The results suggest that long-term repetitive CO_2_ exposure does not increase recovery time significantly (the p-value is 0.09). In both (A) and (B), a LI chip with a 170 µm thick microchamber was used.
(TIF)

Figure S3 Location of the different sets of neurons used in the injury experiments. The ppk-Gal4 driver line, which labels Class IV sensory neurons (depicted in the left schematic in blue), was used to study Ca2^+^ responses to laser ablation of a dendrite (Figure 2). The Class IV sensory neurons were used for this experiment because their cell bodies and their dendrites lie close to the cuticle, allowing for reproducible injury by the pulsed dye laser and excellent visualization of cellular responses close to injury site. aCC and RP2 motoneurons (depicted in the right schematic in green) were used for the study of axonal transport after nerve crush injury (Figure 3) because the regenerative response to injury has been previously characterized in these neurons [22]. The RN2-Gal4 driver line, which labels enteric motoneurons in larvae (depicted in the right schematic in red), was used for time lapse regeneration studies. This driver line is very strong, allowing for both UAS-GMA and UAS-mCD8-RFP to be expressed at high levels. While these neurons display similar reactions to both laser axotomy and nerve crush, we focused upon laser axotomy (Figure 4) because the injury site fits within the field of view of our microscope.
(TIF)

Figure S4 ANF-GFP particle density for on-chip immobilized and flayed-open larvae. In the flay-open protocol, 3^rd^ instar larvae were quickly dissected, mounted between a coverslip and a glass-slide and imaged within 5 min after dissection. The number of anterogradely (gray), retrogradely (blue) and stationary (red) moving particles were analyzed using the on-chip (10 axons) and flay-open methods (24 axons) respectively. No significant differences between the two methods were observed (p-value<0.01).
(TIF)

Figure S5 On-chip imaging of F-actin dynamics after laser axotomy. (a) *In vivo* time-lapse images of the proximal site (PS) of injury, the site of injury (SOI), and the distal site (DS) of injury, 10 hours after laser axotomy, extracted from Movie S6. A single enteric motoneuron was visualized by combining the RN2-Gal4 [48] driver line with UAS-mCD8-RFP (red) to label axonal membrane, and UAS-GFP-moesin [54] (green) to label F-actin. Scale bar, 10 µm. (b) Normalized area change of the proximal stump between 10 and 11 hours after injury.
(TIF)

Movies S1 Laser microsurgery in the SI-chip of sensory neuron dendrites labeled with G-CaMP3.0. UAS-G-CaMP3.0 was expressed in class IV neurons using the ppk-Gal4 driver. Single neurons were imaged by spinning disc confocal microscopy at 5 frames/second. While imaging, a pulsed UV laser was used to transect a primary dendritic branch. The movies were false colored and the scale bar indicates the relative intensity level of G-CaMP3.0. Laser transection induced a rapid increase in G-CaMP intensity, which began at the site of injury and travelled to the cell body.
(WMV)

Movies S2 Laser microsurgery in the SI-chip of sensory neuron dendrites labeled with mCD8-GFP. UAS-mCD8-GFP was expressed in class IV neurons using the ppk-Gal4 driver. Single neurons were imaged by spinning disc confocal microscopy at 5 frames/second. While imaging, a pulsed UV laser was used to transect a primary dendritic branch. The movies were false colored and the scale bar indicates the relative intensity level of mCD8-GFP. mCD8-GFP revealed movements in membranous structures in the cell body, however no intensity increase was observed after dendrite transection.
(WMV)

Movies S3 Axonal transport before injury in the distal and proximal stumps. The movies were taken at 300 ms per frame. Scale bar, 10 µm.
(WMV)

Movies S4 Axonal transport after injury in the distal stump. The movies were taken at 300 ms per frame. In the distal stump, ANF-GFP particles form aggregates and remain stationary 3 hours after injury. Scale bar, 10 µm.
(WMV)

Movies S5 Axonal transport after injury in the proximal stumps. The movies were taken at 300 ms per frame. ANF-GFP particles in the proximal stump move in both directions 3 hours after injury. An increased particle density in the anterograde direction is observed. Scale bar, 10 µm.
(WMV)

Movie S6 Axonal regeneration in motoneurons 7–12 hours after laser injury. RFP was used to label the axonal membrane, GFP-Moesin (GMA) was used to label F-actin [54]. A stack of 5 images was recorded every one minute (exposure time for each image was set to 300 ms). The projection of all images in each stack was used to produce the movie.
(WMV)
