# Supplementary figures and images for: Microfluidic Chips for In Vivo Imaging of Cellular Responses to Neural Injury in Drosophila Larvae
Source: PLoS One. 2012 Jan 23;7(1):e29869. doi: 10.1371/journal.pone.0029869 (PMC3264548; doi:10.1371/journal.pone.0029869)

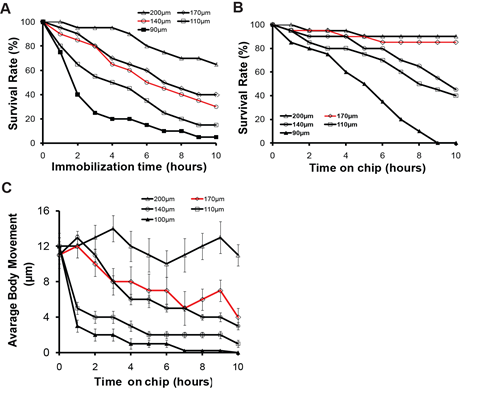

Supplement: Figure S1 — Survival rates and body movement of on-chip immobilized larvae. (A) Survival rate of continuously immobilized larvae using the SI-chip. Five different immobilization microchamber thicknesses were tested. We considered a thickness of 140 µm (red curve) to be optimal, as thicknesses higher than 140 µm resulted in poor immobilization. (B) Survival rates on the LI-chip using periodic immobilization (30 s of immobilization every 5 min). We considered a thickness of 170 µm (red curve) to be optimal as more than 85% of larvae survived the immobilization procedure after 10 hours. (C) Average larva body movement using the LI-chip for different thicknesses of the immobilization microchamber (30 s of immobilization every 5 min). When the mechanical Immobilization was minimized (e.g. using a 200 µm thick microchamber), the average body movement was not affected even after 10 hours of repetitive CO2 application. In all plots, error bars represent standard error of the mean obtained from 10 larvae. (TIF) [file pone.0029869.s001.tif]

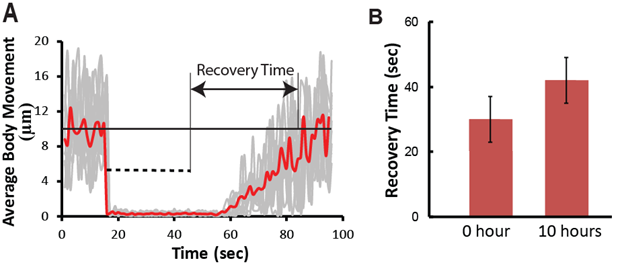

Supplement: Figure S2 — Larva recovery after immobilization on-chip. (A) Larva average body movement before and after the initial immobilization. The red line is the average movement from 10 larvae. The grey lines represent movement from individual larvae. The dashed line represents the immobilization period (30 sec). We define the recovery time as the time needed for the average body movement to reach the pre-immobilization value. (B) Recovery time at the beginning (0 hours) and end (10 hours) of a 10-hour repetitive CO2 immobilization experiment (30 sec CO2 immobilization/5 min resting interval). Error bars represent standard error of the mean. The results suggest that long-term repetitive CO2 exposure does not increase recovery time significantly (the p-value is 0.09). In both (A) and (B), a LI chip with a 170 µm thick microchamber was used. (TIF) [file pone.0029869.s002.tif]

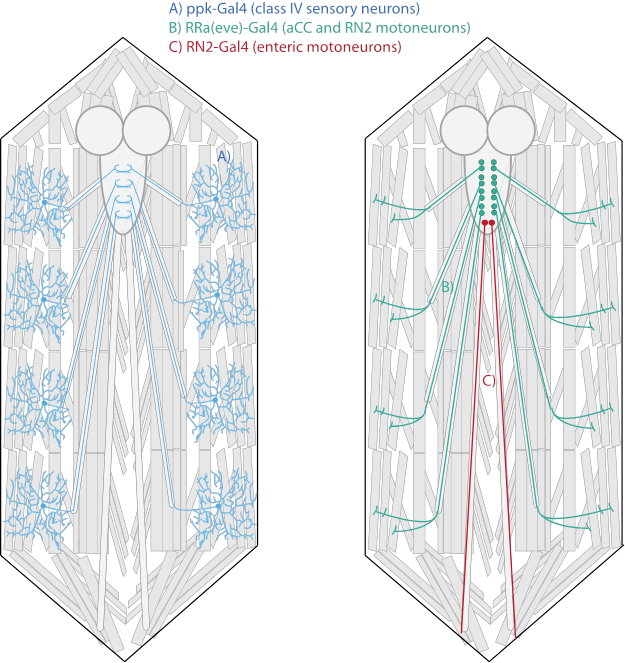

Supplement: Figure S3 — Location of the different sets of neurons used in the injury experiments. The ppk-Gal4 driver line, which labels Class IV sensory neurons (depicted in the left schematic in blue), was used to study Ca2+ responses to laser ablation of a dendrite (Figure 2). The Class IV sensory neurons were used for this experiment because their cell bodies and their dendrites lie close to the cuticle, allowing for reproducible injury by the pulsed dye laser and excellent visualization of cellular responses close to injury site. aCC and RP2 motoneurons (depicted in the right schematic in green) were used for the study of axonal transport after nerve crush injury (Figure 3) because the regenerative response to injury has been previously characterized in these neurons [22]. The RN2-Gal4 driver line, which labels enteric motoneurons in larvae (depicted in the right schematic in red), was used for time lapse regeneration studies. This driver line is very strong, allowing for both UAS-GMA and UAS-mCD8-RFP to be expressed at high levels. While these neurons display similar reactions to both laser axotomy and nerve crush, we focused upon laser axotomy (Figure 4) because the injury site fits within the field of view of our microscope. (TIF) [file pone.0029869.s003.tif]

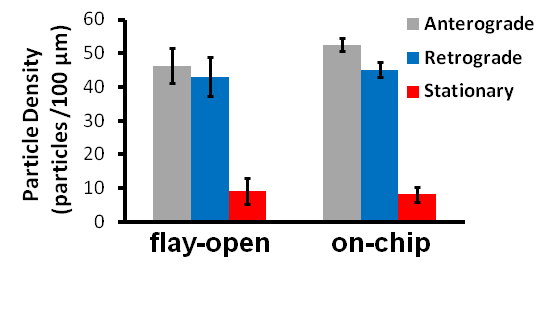

Supplement: Figure S4 — ANF-GFP particle density for on-chip immobilized and flayed-open larvae. In the flay-open protocol, 3rd instar larvae were quickly dissected, mounted between a coverslip and a glass-slide and imaged within 5 min after dissection. The number of anterogradely (gray), retrogradely (blue) and stationary (red) moving particles were analyzed using the on-chip (10 axons) and flay-open methods (24 axons) respectively. No significant differences between the two methods were observed (p-value<0.01). (TIF) [file pone.0029869.s004.tif]

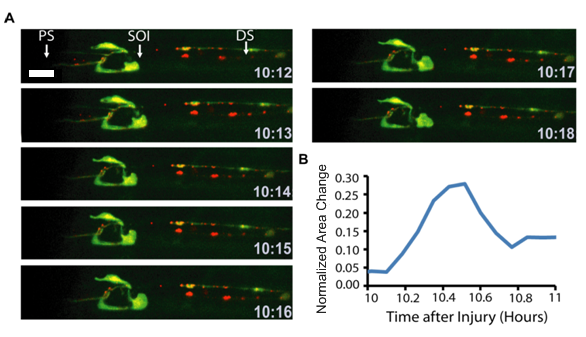

Supplement: Figure S5 — On-chip imaging of F-actin dynamics after laser axotomy. (a) In vivo time-lapse images of the proximal site (PS) of injury, the site of injury (SOI), and the distal site (DS) of injury, 10 hours after laser axotomy, extracted from Movie S6. A single enteric motoneuron was visualized by combining the RN2-Gal4 [48] driver line with UAS-mCD8-RFP (red) to label axonal membrane, and UAS-GFP-moesin [54] (green) to label F-actin. Scale bar, 10 µm. (b) Normalized area change of the proximal stump between 10 and 11 hours after injury. (TIF) [file pone.0029869.s005.tif]
